# Incidental Gallbladder Carcinoma in North Indian Population: Importance of Routine Histopathological Examination of All Benign Gallbladder Specimens

**DOI:** 10.7759/cureus.16156

**Published:** 2021-07-04

**Authors:** Rita Yadav, Mala Sagar, Sanjeev Kumar, Shyam Kumar Maurya

**Affiliations:** 1 Department of Pathology, Vivekananda Polyclinic and Institute of Medical Sciences, Lucknow, IND; 2 Department of Pathology, King George's Medical University, Lucknow, IND; 3 Department of Surgery, King George's Medical University, Lucknow, IND

**Keywords:** incidental gallbladder carcinoma, histopathology, radiology, gall stones, benign gallbladder diseases

## Abstract

Aim: To evaluate the incidence of incidental gallbladder carcinoma in specimens of cholecystectomy performed for benign gallbladder diseases and to establish the utility of routine histopathological examination of all gallbladder specimens obtained following cholecystectomy done for benign diseases.

Material and Methods: This was a hospital-based three years prospective and retrospective study done at a tertiary care centre in North India. Gallbladder specimens from laparoscopic and open cholecystectomies done for benign gallbladder diseases without any clinico-radiological evidence of malignancy were included in the study. Routine histopathological evaluation of 1,268 such gallbladder specimens was carried out to get the incidence of incidental gallbladder carcinoma and pathological staging of carcinoma was done according to American Joint Committee recommendations for cancer staging (AJCC). All the diagnosed cases of Incidental gallbladder carcinoma (IGBC) were analysed in terms of demographics, radiology findings, and gross and microscopic pathology findings.

Results: Out of 1,268 gallbladder specimens of clinically benign diseases, 16 cases (1.26%) were diagnosed as cases of IGBC with female predominance with a male to female ratio of 1:7. Mean gallbladder thickness in these cases was 0.77±0.20 cm, and 98.30% cases of IGBC were associated with gall stone disease. However, no correlation was observed between the age, gallbladder thickness and pathological stages of these IGBC.

Conclusion: IGBC is an occult disastrous malignancy of the gallbladder, which can be missed in the pre and intraoperative periods despite careful clinical and radiological evaluation and comes as a surprise for pathologists the first time. We recommend that all specimens of gallbladder obtained from its surgical resection for benign diseases should be subjected to histopathological examination.

## Introduction

Gallbladder carcinoma is the most common cancer of the biliary tract and the fifth most common cancer of gastro-intestinal tract worldwide and is known for its calamitous course and poor prognosis [1]. While In India, gallbladder carcinoma is the most common cancer of the gastro-intestinal tract [2]. Its geographical and racial distribution is not homogenous, as its highest incidences are reported in Indians, Pakistanis, Chileans, Bolivians, Central Europeans, Israelis, Native Americans and Americans of Mexican origin [3]. In India, its incidence is more along the Gangetic plains of northern India [2,4].

Incidental gallbladder carcinoma (IGBC) is defined as cancer discovered at the time of histopathological examination of the specimen after cholecystectomy done for benign gallbladder disease without clinico-radiological and intraoperative suspicion of malignancy [1,5]. Studies have shown that the incidence of IGBC ranges from 0.2% to 3.3% in all cholecystectomies specimen of benign disease [6]. Gallstone disease is the major risk factor for gallbladder cancer along with other less commonly observed risk factors like calcification of gallbladder wall, adenomatous polyp, obesity, estrogen, choledochal cyst, and chemical carcinogens [1,2,4]. The definitive treatment of symptomatic gallstone disease is a laparoscopic or open cholecystectomy as per the demand of clinical situation and preference of the patient [4]. Keeping in view the incidence of IGBC, every cholecystectomy specimen should be sent for histopathological examination to increase the detection rate of occult early-stage IGBC in benign gallbladder diseases [7]. IGBC has a good prognosis, prolonged survival and potentially curable disease with an adaptation of adequate surgical strategy as compared to non-incidental or primary gallbladder carcinoma [3,5,7]. The use of routine or selective histopathological examination of gallbladder specimens is still debatable.

The aim of this study was to find the incidence of incidental gallbladder cancer in benign gallbladder disease after cholecystectomies specimen and to establish the utility of routine as compared to selective histopathological examination especially in high incidence areas like northern India.

## Materials and methods

This was a descriptive study that analysed all gallbladder specimens of benign diseases received in the department of pathology of our Institution over a period of three years, one year in a prospective manner from April 2020 to March 2021 and two years in a retrospective manner from April 2018 to March 2020. Written informed consent was taken from all the participants of the study. Inclusion criteria were all laparoscopic and open cholecystectomy specimens of benign gallbladder diseases which were clinico-radiological unsuspected cases of gallbladder cancer and diagnosis of gallbladder cancer was made for the first time on histopathological examination. Exclusion criteria were patient’s age under 18 years and clinico-radiological suspected cases of gallbladder cancer. Diagnosis of IGBC was made on formalin-fixed, paraffin-embedded, hematoxylin and eosin-stained sections and pathological staging of carcinoma was done according to American Joint Committee recommendations for cancer staging (AJCC). The gallbladder wall was said to be thickened if it was found to be >3 mm on preoperative imaging or histopathological examination. The normal thickness of the gallbladder wall is reported to be 1-2 mm. All the diagnosed cases of IGBC were analysed and data on demographics, radiology findings (USG and CT-scan), gross and microscopic pathological findings were collected from the radiology electronic database and laboratory request forms.

## Results

Total 14,876 biopsy specimens from different organs were received in the Department of Histopathology over a period of three years. Out of which 1,268 (8.52%) were specimens of gallbladder obtained from cholecystectomy done for clinically and radiologically established benign gallbladder disease. In all these cases, the treating clinician and a radiologist were not suspicious of any kind of gallbladder malignancy despite complete clinical and radiological evaluation of the patient. We performed a microscopic examination of all these cases and observed that among the all benign specimens 16 cases (1.26%) were having evidence of IGBC, which was the first time diagnosed by histopathological examination without clinico-radiological suspicion of malignancy (Table 1).

**Table 1:** The incidence of IGBC IGBC - Incidental gallbladder carcinoma

Finding	N	%	95% CI for incidence
Total biopsy	14,876		-
Benign gallbladder	1,268	8.52	(8.08-8.97)
Incidental gallbladder carcinoma	16	1.26	(0.65-1.88)

**Figure 1:**
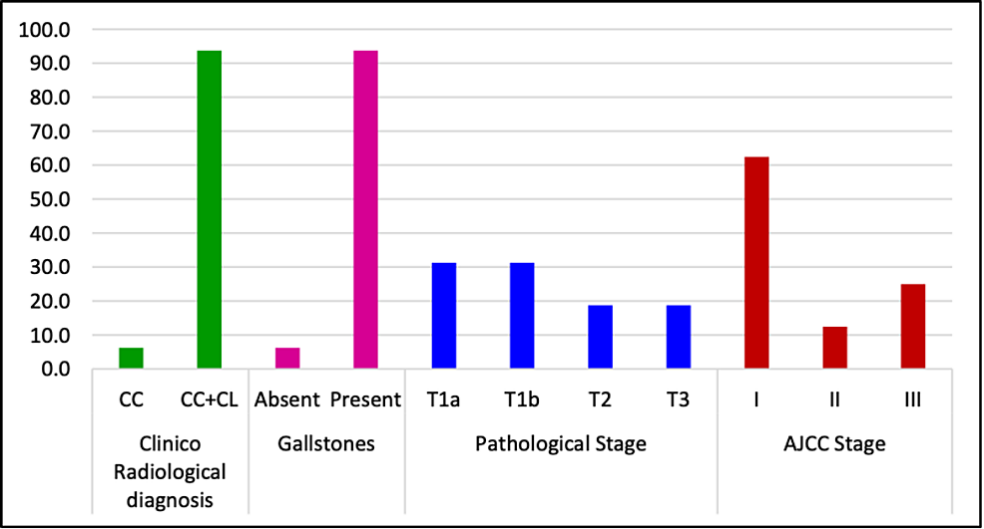
The clinico-pathological spectrum of cases of IGBC IGBC - Incidental gallbladder carcinoma CC - Chronic cholecystitis CL - Cholelithiasis AJCC - American Joint Committee recommendations for cancer staging

Analysis of demographic profile and clinico-pathological features of these cases are summarised in Tables 2, 3 and Figure 1. There was female predominance (M:F ratio 1:7) among cases of IGBC in our study (Table 1). This can be attributed to the female hormone oestrogen, which increases the chances of cholelithiasis more in females. The age of the patients ranged from 40 to 78 years (mean 58.63±9.99 years) (Table 2). The mean wall thickness of the gallbladder was 0.77±0.20 cm (Table 2). A preoperative abdominal ultrasound was done in all the cases and 93.8% cases were associated with gall stones.

**Table 2:** The distribution of cases according to sex, age and wall thickness

Variable		Value
Sex	Male N(%)	2 (12.5)
	Female N(%)	14 (87.5)
Age (Mean±SD)		58.63±9.99
Mean wall thickness (Mean±SD)		0.77±0.20

 

**Table 3:** The clinico-pathological features of cases CC - Chronic cholecystitis CL - Cholelithiasis AJCC - American Joint Committee recommendations for cancer staging

Diagnosis	Status	N (%)	Percent
Clinico-radiological diagnosis	CC	1 (6.3)	6.3
	CC+CL	15 (93.8)	93.8
Pathological stage	T1a	5 (31.3)	31.3
	T1b	5 (31.3)	31.3
	T2	3 (18.8)	18.8
	T3	3 (18.8)	18.8
AJCC stage	I	10 (62.5)	62.5
	II	2 (12.5)	12.5
	III	4 (25.0)	25.0

 

On pathological staging, most of the incidentally detected gallbladder cancers were found to be in surgically resectable stages like T1a (31.3%), T1b (31.3%) and T2 (18.8%). The AJCC stages I, II and III were in proportion 62.5%, 12.5% and 25.0%, respectively.

The pathological stage was higher with an increase in age; however, no significant association of age was found with the stage (p=0.068) (Table 4). Moreover, gallbladder wall thickness was also not significantly associated with the pathological stage of the tumour (Table 5).

**Table 4:** The association of age with pathological stages

Stage	N	Age in years		F-value	P-value
		Mean	SD		
T1a	5	53.2	10.0	3.092	0.068
T1b	5	55.8	6.6		
T2	3	71.0	10.4		
T3	3	60.0	5.0		

**Table 5:** The association of wall thickness with pathological stages

Stage	N	Mean wall thickness		F-value	P-value
		Mean	SD		
T1a	5	0.8	0.3	1.496	0.266
T1b	5	0.7	0.1		
T2	3	0.9	0.2		
T3	3	0.8	0.1		

## Discussion

The association of wall thickness with pathological stages IGBC is defined as carcinoma of gallbladder diagnosed first time on histopathological examination after cholecystectomy done for benign gallbladder disease without clinico-radiological and intraoperative suspicion of malignancy [4,5]. The incidence of IGBC is reported to be 0.2% to 3.3% of all cholecystectomies specimen of benign gallbladder disease [5,6,8-11]. This range is due to the geographical and racial heterogenous distribution of gallbladder malignancies worldwide [3]. In our study, IGBC was found to be 1.26% and our results are in the agreement with some studies from India and Nepal [9,10]. However, various studies from North India have shown variation in incidence of IGBC (Table 6). This variation in the incidence of IGBC in the north Indian population may be due to different lifestyles, cultural and dietary factors.

**Table 6:** The incidence of IGBC as reported by various studies

S.N.	Study	Year	Place of study	Incidence
1.	Our study	2021	North India	1.26%
2.	Sangwan et al. [9]	2015	North India	1.9%
3.	Butti et al. [2]	2020	North India	0.77%
4.	Jha et al. [1]	2018	North India	0.41%
5.	Poudel et al. [11]	2020	Nepal	1.67%
6.	Ghimire et al. [10]	2011	Nepal	1.28%
7.	Mauro et al. [6]	2021	U.K.	0.1%

In our study, there was female predominance (M: F ratio 1:7) and the mean age of the patients is 58.6 years, which was in concordance with the previous literature [1,2,12,13]. The female predilection of IGBC may be explained by the fact that the female hormone estrogen increases the saturation of cholesterol in bile and thus increases the possibility of gall stone diseases. Cholelithiasis is a major risk factor for gallbladder cancer [2]. We found that cholelithiasis was associated with 93.7% cases of IGBC in our study, which is almost similar to the study of Gulwani et al. who found that 95.6% cases of IGBC were associated with gall stone disease [4]. Clinical manifestation of IGBC is non-specific and similar to various benign gallbladder diseases; hence, it is difficult to diagnose clinically and at times it presents as a surprise to pathologists [3].

The normal thickness of the gallbladder wall is 1-2 mm. On macroscopic histopathological examination, if the thickness of the gallbladder wall is >3 mm, then it is called thickened gallbladder wall [12]. The gallbladder wall thickening is a nonspecific finding that may be associated with a wide range of gallbladder diseases like acute cholecystitis, chronic cholecystitis, cholelithiasis and malignancies [13]. Hence, occult carcinoma gallbladder or IGBC is almost impossible to be diagnosed on radiology and macroscopic examination especially on the basis of gallbladder wall thickness [12,14, 15]. The only way to diagnose the early stage of gallbladder cancer or IGBC is microscopic histopathological examination [3,9]. In our study, the mean wall thickness of the gallbladder was 0.77±0.20 cm and IGBC was detected in all cases of thickened gallbladder wall on microscopic histopathological examination, which was preoperatively diagnosed as cases of chronic cholecystitis and cholelithiasis by radiologists and surgeons. The present study highlights the fact that it is difficult to suspect malignancy in gallbladder specimens on clinico-radiological examination. We recommend all the benign gallbladder specimens should be sent for histopathological examination after cholecystectomy.

In the literature, there has been debate on routine versus selective histological assessment of benign gallbladder specimens after cholecystectomy. Most of the studies in the literature recommend that the routine histopathological examination of all post-cholecystectomy specimens is the safest approach to increase detection of IGBC [5,7,16-18]. Torres et al. recommended that all the benign gallbladder specimens must be sent for routine histopathological examination in order not to miss IGBC [5]. When using a selective approach of histopathological examination of cholecystectomy specimens, it is important to make a meticulous on-table evaluation of the specimen to reduce the risk of missing the IGBC [5].

Lundgren et al. concluded that IGBC detected in high proportion when performed a routine histopathological examination of all post-cholecystectomy specimens [16]. Using a selective approach misses some of the IGBC cases because a macroscopic examination is not sufficient to exclude gallbladder malignancy [16].

Jaysundera et al. opined that a selective approach for gallbladder histopathology may be an option in geographical areas having a low incidence of gallbladder carcinoma but advocating it as a universal approach is not justified [17]. In the countries or subcontinents having a high prevalence of gallbladder carcinoma like Italy, India, Nepal and Pakistan, it is recommended that all gallbladder specimens should be subjected to routine histopathological examination so that cases of IGBC are not missed [17]. Recent studies from India suggested that the actual incidence of IGBC in our country may be much higher than reported in literature and avoidance of routine microscopic examinations of gallbladder specimens may be dangerous and also, missing an IGBC is an unaffordable affair [7].

Royal College of Pathologist also suggests that all gallbladder specimens are mandatory to submit for histopathological examination because many significant pathologies can present with normal morphological appearance [18].

Nevertheless, there are studies that discourage the routine histopathological examination of all gallbladder specimens stating that it is unnecessary to put a burden of cost and workload on pathologists and they suggested a selective approach of histopathological examination of cholecystectomy specimen [6,8,15].

In this context, Tayeb et al. performed a macroscopic and microscopic examination of all gallbladder specimens obtained from cholecystectomy done for gallbladder stone diseases and they found that the incidence of IGBC was 0.7%, which was associated with at least some abnormality in macroscopic examination and not a single IGBC was detected in grossly normal-looking gallbladder [8]. Hence, they concluded that selective histopathological examination should be done only for the cases, which show any kind of abnormality in the gross examination.

Emmet et al. advocate the selective approach of histopathological examination but at the same time, they recommended that the gallbladder specimen from patients having a risk factor for carcinoma like older age, Asian and African patients, gall stone disease, female gender, chronic inflammation, raised alkaline phosphate levels prior to surgery, should be subjected to histopathological examination [15].

Treatment and prognosis of gallbladder carcinoma depend on its pathological stage [11,12]. Non-IGBC usually presented itself in the advanced stages and carries a poor prognosis [10]. IGBC is usually detected in early stage on histopathological examination and carries a good prognosis [5]. In our study, most of the patients were in an early pathological stage like T1a and T1b (62.6%) and carries a good prognosis.

Simple cholecystectomy is recommended for stages Tis and T1a IGBC, which provide a five-year survival rate of almost 100%. However, radical re-operation is recommended for stages T1b, T2 and T3 carcinoma [19]. Radical re-operation should be performed as soon as possible once the final histopathological report is available. But it can also be done in patients who are present after two months of index cholecystectomy [5].

## Conclusions

IGBC is an occult but calamitous malignancy of the gallbladder, which is usually diagnosed for the first time by the pathologist in most cases while performing a routine histopathological examination of benign gallbladder specimens. The incidence of IGBC at a single tertiary care hospital was found to be 1.26%. Despite vigilant clinical and radiological examination, cases of IGBC can be missed and presents as a surprise to histopathologists. We recommend that routine microscopic histopathological examination of benign gallbladder specimens should be performed specially after cholecystectomy in high incidence areas.
